# PREDICTING SOCIAL ISOLATION IN OLDER ADULTS WITH COGNITIVE DECLINE

**DOI:** 10.1093/geroni/igae098.4049

**Published:** 2024-12-31

**Authors:** Bada Kang, Min Kyung Park, Jennifer Kim, Seolah Yoon, Seok-Jae Heo, Chaeeun Kang, Dahye Hong

**Affiliations:** Yonsei University, Seoul, Republic of Korea; Yonsei University College of Nursing, Seoul, Republic of Korea; Yonsei University, Seoul, Republic of Korea; Yonsei University, Seoul, Republic of Korea; Yonsei University College of Medicine, Seoul, Republic of Korea; Yonsei University, Seoul, Republic of Korea; Yonsei University, Seoul, Republic of Korea

## Abstract

Social isolation in older adults, which encompasses limited social interaction and loneliness, is a significant risk factor for dementia. Subjective cognitive decline (SCD) and mild cognitive impairment (MCI) stages are considered focal points of preventive interventions, at which cognitive and physical functions can be restored. Thus, preventing social isolation via prompt identification of high-risk individuals is crucial. This study aimed to develop and validate machine learning models to predict social interaction and loneliness levels among older adults with SCD and MCI. This study included 99 community-dwelling older adults (n=67 SCD and 32 MCI). While demographic and health-related data were collected via survey, we applied a mobile Ecological Momentary Assessment approach for real-time measurement of social interaction and loneliness levels and wrist-worn actigraphy for sleep and activity data. While the Random Forest model was the most suitable for predicting social interaction level (area under the receiver operating characteristic curve [AUC]: 0.935), the Gradient Boosting Machine model was the most suitable for predicting high levels of loneliness (AUC: 0.887). Time-specific analyses demonstrated the association between a low frequency of physical movement in the morning and a low level of social interaction, and decreased sleep quality during night time and high levels of loneliness. This study showed the potential of machine learning models to predict the social interaction and loneliness of older adults at risk for dementia. The algorithms can be applied to develop mobile preventive interventions that utilize real-time sleep and behavioral data to prevent social isolation in these at-risk individuals.

